# Senecio Aureus

**Published:** 1845-06

**Authors:** 


### SELECTIONS

FROM

### AMERICAN AND FOREIGN JOURNALS.

---

*Senecio Aureus.*—Class Syngenesia, Superflua. Life-root, Gravel-root, by the Indians Nun-qua. This plant is found only in swamps and the vicinity of sluggish streams, growing in black muck, and may generally be looked for in the neighborhood of the Caltha palustris, which it somewhat resembles, the leaf being smaller and less succulent. Commonly but two leaves grow from the same root, on long petioles (6 to 12 inches), one leaf on each, serate and ovate-cordate. In May, it sends up a purplish flower stalk from 18 to 24 inches high, the leaves of which are pinnatified and toothed. The flowers are bright yellow, resembling the Taraxacum, and generally three or four in a sort of umbel. The plant remains green all winter, and as the young leaves start up in the spring the old ones decay. It has a small purplish horizontal root, lying near the surface, sending off minute radicles into the soft soil beneath. The whole plant is used, and is easily and rapidly gathered without digging, being pulled up, by clasping the horizontal portion.

Given in infusion it promotes perspiration, the secretion of urine—increases the force of the circulation, without producing any febrile tendency, and is particularly useful in those cases of anemia, attended with cold extremities, feeble circulation and inability to endure muscular exertion. In dysmenorrhea, a cupful of the warm infusion frequently gives entire relief, abating the pain and procuring a free flow of the catamenial secretion. It is highly useful in all cases, where a diaphoretic and diuretic agent is required, and more especially if the case may be attended with spasm of the urinary or uterine systems. It seems to exert an antispasmodic influence over the capillary vessels, when the circulation is languid, enabling them to relieve themselves of accumulations of venous blood, imparting energy and power to the whole circulatory apparatus.—*N. Y. Jour. of Med.*

